# Clinical Applications of Immunotherapy Combination Methods and New Opportunities for the Future

**DOI:** 10.1155/2017/1623679

**Published:** 2017-08-07

**Authors:** Ece Esin

**Affiliations:** Dr. A. Y. Ankara Oncology Research and Training Hospital, Ankara, Turkey

## Abstract

In the last decade, we have gained a deeper understanding of innate immune system. The mechanism of the continuous guarding of progressive mutations happening in a single cell was discovered and the production and the recognition of tumor associated antigens by the T-cells and elimination of numerous tumors by immune-editing were further understood. The new discoveries on immune mechanisms and its relation with carcinogenesis have led to development of a new class of drugs called immunotherapeutics. T lymphocyte-associated antigen 4, programmed cell death protein 1, and programmed cell death protein ligand 1 are the classes drugs based on immunologic manipulation and are collectively known as the “checkpoint inhibitors.” Checkpoint inhibitors have shown remarkable antitumor efficacy in a broad spectrum of malignancies; however, the strongest and most durable immune responses do not last long and the more durable responses only occur in a small subset of patients. One of the solutions which have been put forth to overcome these challenges is combination strategies. Among the dual use of methods, a backbone with either PD-1 or PD-L1 antagonist drugs alongside with certain cytotoxic chemotherapies, radiation, targeted drugs, and novel checkpoint stimulators is the most promising approach and will be on stage in forthcoming years.

## 1. Introduction

*“Natural forces within us are the true healers of disease”* is a famous quote from Hippocrates which refers to the recent renaissance of cancer treatment. In the last decade, we have gained a deeper understanding of innate immune system and T-cell recognition. In particular, researchers found that the ignorance of self-proteins, which protect the body from autoimmune diseases (the* “natural forces”* in Hippocrates quote), acts as a key mechanism behind tumoral escape from destruction. Additionally, the mechanism of the continuous guarding of progressive mutations happening in a single cell (immune-surveillance) was discovered; the production of new cancer cell antigens (neoantigens), the recognition of both cancer specific and malignancy associated antigens by the T-cells, and elimination of numerous tumors by immunoediting were understood in detail. The new discoveries on immune mechanisms and its relation with carcinogenesis have led to development of a new class of drugs called immunotherapeutics (IT).

Cancer cells create an immunosuppressive microenvironment and grow inside it. In normal conditions, the immune system is capable of distinguishing the danger signal and capable of inducing an appropriate reaction towards tumor cells. The tumor associated antigens are recognised by T-cells, which leads to tumors being eradicated; however, tumoral cells escape from immunoediting by expressing programmed cell death ligand (PDL-1) and similar inhibitory gene products like IDO (indolamine 2,3 dioxygenase), TGF-*β* (transforming growth factor-*β*), and Interleukin-10 (IL-10). One of the mechanisms of cancer evolution to escape from antitumor guarding of immune system is the deactivation or silencing the effector T-cells. T-cell exhaustion is mediated by inhibitory receptors such as programmed cell death protein-1 (PD-1), TIM-3 (mucin 3), and LAG-3 (Lymphocyte activation gene protein-3). One of the major cytokines released from T-cells, Interferon-*γ* (IFN-*γ*), creates a vicious cycle of immunosuppression by increasing PD-1 expression.

Despite the promising developments, the strongest and most durable immune responses do not last long, as resistance eventually develops and the more durable responses only occur in a small subset of patients. From the knowledge and experience of classical cytotoxic drugs, one of the solutions which have been put forth to overcome the challenges encountered in clinical practice is combination strategies.

## 2. The Rationale and Scientific Background of Combinatorial Immunotherapies

The idea that cancer treatment can occur via induction of immune response has been studied for more than a century [1, 2]. First hypothesis of immunotherapy relies on William Coley's, the father of immunotherapy, studies [3–5]. Coley experienced the beneficence of* Streptococcus* pyogenes infection in an inoperable sarcoma patient who obtained complete remission. Based on the foresight of an immunological efficacy of* Streptococcus* pyogenes infection against tumor cells, he treated nearly 100 mixed type cancer patients with a 10% overall response rate [4, 5]. While the mechanism of action was not known at that time, it is well known now that activation of immunologic response is based on leucocyte infiltration, clonal increase in T-cell population, and the increase in the release of inflammatory molecules which are the intermediary steps of the efficacy Coley's toxin [3, 4, 6, 7]. However, until very recently, definitive agents of immune manipulation have not been obtained apart from interleukin and interferon approaches in melanoma and metastatic renal cell cancer (mRCC) [8–10]. On the other hand, translational research on immunotherapy has given results, which brought the antagonists of cytotoxic T lymphocyte-associated antigen 4 (CTLA-4), programmed cell death protein 1 (PD-1), and programmed cell death protein ligand 1 (PDL-1) to clinic [11, 12]. Two classes of most widely and effectively used drugs based on immunologic manipulation are collectively known as the “checkpoint inhibitors.” CTLA molecule specifically inhibits T-cell activation and proliferation by binding to CD80 and CD86 and by suppressing costimulatory receptor CD28 and intracellular signaling [13]. PD-1 molecule is a transmembrane protein expressed mainly on T-cells, B cells, and natural killer (NK) cells which exhibits its inhibitory function by binding to specific receptors such as PD-L1 on tumor cells, various tissues, and PD-L2 on hematopoietic cells. The lock-and-key interaction leads to inhibition and T-cell exhaustion, which enables tumor cell to evade from active immune system guarding of cancer cells [14, 15].

CTLA-4 blockage was first tested in melanoma cases with an anti-CTLA-4 inhibitory molecule ipilimumab [16–18]. Ipilimumab was the first proven drug that demonstrated improved survival advantage in metastatic melanoma [16, 17]. Beyond the advantage of survival, complete responses have been obtained, and a plateau has been achieved in the survival curve which never occurred before in melanoma trials except for a limited number of patients [19]. The encouraging results of ipilimumab in melanoma have been supported by trials with a PD-1 antagonist, and overall advantage of survival in addition to improvement in objective response rate [20] and progression free survival (PFS) were shown in randomized controlled phase III trials of PD-1 antagonists Nivolumab and pembrolizumab [21–25]. Further trials with PD-1 inhibitory molecules have been run in melanoma and in other various tumors like RCC, nonsmall cell lung carcinoma (NSCLC), bladder cancer, and others [12, 25–30]. Checkpoint inhibitors have shown remarkable antitumor efficacy in a broad spectrum of malignancies and even in some refractory cases [31–33]. However, despite these promising results and the characteristic response durability, ipilimumab, nivolumab, pembrolizumab, and atezolizumab (a PD-L1 antagonist agent approved for advanced urothelial tumors by FDA in 2016) as single agents only have a range of 10–35% response rates. Only a small number of patients have benefited from immunotherapeutics.

The next challenge for scientists has been to enhance and broaden the overall benefits of IT. Predictive biomarkers like the PD-L1 expression on tumor tissue, mutational load of specific cancer type, and genetic signatures for inflammation have been put forward as a solution for issues with patient selection. Apart from patient selection, knowledge from cytotoxic drugs of cancer leads to the idea that the combination of immunotherapy drugs might allow blockage of different mechanisms of tumor development, overcome resistance, and improve response rates to increase the proportion of patients who benefit from the treatment. This review focuses on combination strategies of anti-CTLA-4, anti-PD1, and PD-L1 molecules with other coinhibitory molecules, costimulatory molecules, agents for molecules in tumor microenvironment, experimental cancer vaccines, cytotoxic chemotherapeutics, targeted agents, and radiation (Table 1).

## 3. Combination Strategies

### 3.1. Combinatorial Immunotherapies with Checkpoint Inhibitors

CTLA-4 inhibitors and PD1 blockers act differently by blocking parallel but distinct pathways on tumor cells. Although both of the molecules have similar negative input on T-cell activity, the timing of downregulation and the anatomic positions of action differ. These pathways operate on different stages of immune reaction. CTLA-4 is considered to be the chief of checkpoint inhibitor orchestra which has a role of stopping autoreactive T-cells in lymph node at initial priming stage [34, 35]. CTLA-4 molecule prevents T-cell activation and proliferation, and it blocks intracellular signaling by preventing the bonding of B7 ligands to T-cell costimulatory molecules via binding to CD80 and CD86 [13, 32, 35]. On the other hand, PD-1 is located on the T-cell surfaces and functions during the effector phase and the PD-1 pathway operates on later stages in peripheral tissues by regulating activated T-cells. Upon recognition of T-cell activation, it binds to PD-L1 and PD-L2 receptors, which results in T-cell exhaustion [14, 15, 36].

Preliminary results showed that the combined administration of ipilimumab and nivolumab results in an enhanced level of antimelanoma activity, compared to monotherapy with either agent with the cost of increased immune-related adverse events [24, 37, 38]. In the first dose escalation study of ipi-nivo combination in melanoma, 53 patients received concurrent treatment while 33 patients received sequential treatment. In the concurrent arm, 65% clinical benefit rate was among the best results of a melanoma trial [37]. The response was remarkably durable, strong, and rapid. In the subsequent phase II trial of ipi-nivo combo, 59% clinical benefit rate was achieved with improved durable results [38]. Wolchok et al. has designed a phase III trial of ipi-nivo combo for treatment of naive melanoma patients and showed that overall response rate (ORR) was 57% with combination compared to only 19% response rate (RR) in ipi alone arm and 43% in Nivo-only arm. The updated results of CheckMate 067 trial showed that PFS 11.5 months was improved with a hazard ratio (HR) of 0.42 in combination arm (11.5 m) against the ipi-only arm (2.9 m) [39]. It is well described that combination strategy with dual checkpoint inhibition has remarkably improved the outcomes of patients. The CheckMate 204 trial was design to show efficacy of ipi + nivo combination especially in asymptomatic brain metastatic melanoma patients [40]. The primary endpoint was intracranial (IC) response rate and the results showed that IC response rate was 56%, and 19% of patients had a complete response. Not surprisingly, IC and extracranial responses were found to be largely concordant. Grade 3/4 AEs occurred in 48% of patients, 8% neurologic, including headache and syncope. Only 3 patients (4%) stopped treatment due to therapy related neurologic toxicity.

The second method of the in-group combination of checkpoint inhibitors is with pembrolizumab and ipilimumab. KEYNOTE-029 (NCT02089685) was a phase 1/2 study designed to assess the safety and efficacy of pembro + ipi in patients with advanced melanoma or RCC. According to the results of phase 1b of Keynote-029 trial pembrolizumab plus low-dose ipilimumab was tolerable and effective for patients with advanced melanoma, with an overall response rate (ORR) of 56%. Very recently, Matteo et al. presented the mature data of Keynote-029 trial which estimated 1-year PFS as 69% and 1-year OS as 89% [41]. As far as the safety concerned, immune-mediated AEs occurred in 90 (59%) patients; 25% were grade 3/4 and no treatment-related (TR) deaths occurred. In an Australia trial, the ABC trial, same strategy was tested for asymptomatic brain metastatic melanoma patients without previous cranial therapy [42]. PFS for 6 months was 50% in combo arm versus 29% in nivo alone arm; similarly 6-month OS was 76% versus 59%. Treatment-related grade 3/4 toxicity was reported as 68% versus 40%.

The encouraging results from the melanoma trials have led to the exploration of the use of this combination in other malignancies. Hammers et al. studied ipi-nivo combination in 2 different dose scale (Nivo 1 mg/kg versus 3 mg/kg + ipi 3 mg/kg versus 1 mg/kg) in mRCC. Similar results were observed as in melanoma trials, with up to 40% ORR. Furthermore, 65% of patients were progression-free at 24 weeks; however, grade 3 adverse events were in 62% of study population in nivo1ipi3 arm, and there was a 6/47 treatment discontinuation in nivo1ipi3 arm due to treatment-related adverse events (TAEs) [43]. The phase III trial of Ipi-nivo combination against sunitinib in previously untreated mRCC patients has recently been completed and results will be determined in 2019 (NCT02231749).

Nonsmall cell lung cancer is another malignancy where immunotherapy has reshaped the treatment landscape. Nivolumab is a FDA approved agent in both squamous and nonsquamous NSCLC that experience progression of disease on or after standard platinum-based chemotherapy (regardless of tumor PD-L1 protein expression). In CheckMate 057 trial, 3-month overall survival benefit was shown in nivo arm against docetaxel in second line (HR 0.72, 95% CI 0.60–0.88) in nonsquamous NSCLC [44]. In CheckMate 017 trial, 3-month overall survival benefit was again shown with HR of 0.59 (95% CI 0.44–0.79) [45]. An initial study of ipi-nivo combination showed some level of activity (16% ORR) with high grade of toxicity (35% treatment discontinuation) [46]. CheckMate 227 trial is a phase III study testing ipi-nivo combo in stage IV NSCLC and is currently recruiting participants.

Two other checkpoint inhibitor molecules, tremelimumab (Tre, CTLA-4 inh.) and durvalumab (Dur, PD-L1 inh.) have been studied in NSCLC both as single agents and in combination [47]. Ten different dose escalation cohorts were tested and higher TAEs were observed with increased tremelimumab doses. ORR were as high as 33% with manageable toxicity profile in lower dose cohorts of tremelimumab. As a result of promising ORR in NSCLC of this study, the 20 mg/kg durvalumab + 1 mg/kg tremelimumab combination was chosen as a result for further phase II and III studies. NCT02453282 study is a phase III study which is comparing tre-dur combination against tre monotherapy completed patient accrual and is estimated to be reported at 2018. In sum, combination strategies in NSCLC have yielded encouraging result and will continue to be investigated further in various studies, highlighting the position of immunotherapy in the general landscape of NSCLC treatment.

The use of CTLA and PD-1 combination is not limited to melanoma, RCC, and NSCLC. A growing body of literature shows that this combination is effective in other malignancies as well. Antonia et al. have showed that ipi-nivo combo can have durable antitumoral response with manageable toxicity in previously treated platin-resistant small cell lung cancer (SCLC) [48]. Furthermore, several clinical trials are already recruiting patients in various tumor types including gastric cancer, head and neck cancer, sarcoma, and endometrial carcinoma, and combinations are being tested in basket trials (NCT02872116, NCT02982486, NCT01658878, and NCT02304458).

Lymphocyte activation gene-3 (LAG-3) is a coinhibitory molecule which enhances regulatory T-cell activity and inhibits T-cell proliferation and effector function [49, 50]. T-cell immunoglobulin and mucin domain 3 (TIM-3) is an inhibitory receptor under the control of helper T-cells and cytotoxic T-cells via IFN-*γ* [51]. Higher expression of TIM-3 was found to be associated with T-cell exhaustion [52]. In preclinical models, monotherapy with LAG-3 or TIM-3 blockade resulted in antitumoral activity and synergistic effect with PD-1 and PD-L1 blockade [53, 54]. There are ongoing preclinical and clinical trials to study the synergism of LAG-3 and TIM-3 inhibition with checkpoint inhibitors.

In sum, single agent durable responses of CTLA-4 inhibitors, PD-1 antagonists, and ORR in almost 25% of patients provide a strong rationale for checkpoint inhibitors being used as backbone in combination immunotherapy regimens.

### 3.2. Combinatorial Immunotherapies with Checkpoint Stimulators

Apart from the targeting invisibility of tumor cells by the immune system, another target for developing immunotherapy are the activator pathways of innate immunity. In murine models, 3 molecules were found to be effective as treatment strategy goals: OX40 (tumor necrosis factor receptor superfamily member 4), GITR (glucocorticoid-induced tumor necrosis factor receptor-related protein), and 4-1BB (CD137). OX40 is a secondary stimulatory molecule expressed by activated T-cells and is responsible for T-cell expansion, activator signal expression, and inhibition of regulatory T-cells [55–57]. OX40 agonism via selectively designed antibodies has showed antitumor response and has been tested in combination with PD-1 antagonists, which yielded promising results [58, 59].

GITR is another stimulatory surface protein responsible for regulatory T-cell suppression and creates resistance by regulatory T-cell inhibition. Both preclinical and in vivo models have showed that GTIR agonism results in reduction in the regulatory T-cell accumulation within tumoral tissue [60, 61]. Dual therapy with GTIR agonism and anti PD-1 inhibition was tested in murine models [62] and resulted in clinical activity as dual therapy, which lead to development of further clinical trials (NCT02221960, NCT01239134) [60, 61].

The surface protein 4-1BB is a multistimulatory receptor protein primarily expressed in T-cells, NK cells, and regulatory T-cells [63]. 4-1BB stimulation leads to an enhancement of the activity of cytotoxic T-cells and increase in survival rates [64]. Murine models showed that 4-1BB is a targetable agent that leads to immune activation and clinical response [65]. A 4-1BB agonist antibody Urelumab was tested in Phase I basket trial, where melanoma patients showed clinical response but at the cost of significant liver toxicity [66].

The strategies of inhibition of the checkpoint with PD-1 and the activation of costimulatory molecules with specific agonistic antibodies are complementary to each other and showed synergistic effects in previous trials, thus providing a compelling rationale for further combination trials.

### 3.3. Combination of Immunotherapeutics with Cancer Vaccines and Oncolytic Viruses

Oncolytic viruses offer synergistic effects with checkpoint blockade by inducing immunogenic cell death and inflammatory tumor response. The use of immune-based treatment approaches is expected to rise, with an increase in variety of the approaches. In preclinical models, Newcastle disease virus (NDW) injections have resulted in systemic responses, and together with anti CTLA-4 therapy, the overall response rates in NDW injections have increased [67, 68].

Talimogene laherparepvec (T-VEC) is an oncolytic virus therapy generated from herpes simplex virus-1. OPTIM study, when compared to T-VEC with a nonstandard treatment arm (GM-CSF), has had durable response rates [69]. After that, T-VEC and ipilimumab combination was tested in phase Ib trial, in which 56% RR was observed [70]. Further studies are needed to conclude on the benefit of combination treatment approach of T-VEC with other IT.

### 3.4. Combinatorial Immunotherapies with Cytotoxic Chemotherapy

Over the past decades, substantial evidence has been found supporting the idea that cytotoxic chemotherapy agents may have potential immune modulatory actions besides being active during cell division and inducing apoptosis. The interaction between the chemotherapeutics and immune system resembles a commensalism. The presence of tumor infiltrating lymphocytes is associated with increased response of CT, whereas some agents like gemcitabine, paclitaxel, cyclophosphamide, and 5-fluorouracil may improve immunity by suppressive T-cell depletion and cytotoxic T-cell activation [71–74]. The findings are consistent with the outcomes from clinical trials. In the first line treatment with NSCLC and SCLC, ipilimumab was used with carboplatin/paclitaxel [75, 76]. Although the response rates were shown to be similar to historical controls, durable responses might occur, which might warrant further clinical trials.

One of the most popular topics of cytotoxic drug and immunotherapy combo trials is gastrointestinal system and especially colorectal cancer (CRC). When considering genomic instability across tumor types, CRC stay in the middle of row in terms of mutational load; however there is heterogeneity. A subset of CRC possesses markedly elevated mutational burden; predominantly these types of CRC are characterized by high microsatellite instability [77, 78]. Nowadays there is an ongoing effort to classify the colorectal cancers according to genomic profiles [79]. Four consensus molecular subtypes (CMS) of CRC were defined upon agreement [80]. The CMS 1 subtype is characterized by hypermutation, microsatellite instability, and strong immune activation especially [80]. CMS 2 and CMS 3 tumors show low inflammatory and immune characteristics and CMS 4 tumors demonstrate inflammatory and immunosuppressive signatures. Hence, different strategies and different catalyzers and combinations may be required for the success of immunotherapy in subtypes. Microsatellite instability may be a biomarker for immune response to chemotherapy and immunotherapeutic synergy [77]; however, optimal dosing, optimal timing, and necessary precautions to avoid adverse events need to be investigated.

Classical cytotoxic drugs act on tumor microenvironment and create immunogenicity via therapy-induced cell death. Both 5-FU and oxaliplatin have been thought to have a beneficial effect [81]. Based on this hypothesis, FOLFOX is being combined with pembrolizumab in two studies, targeting GI cancers or colon cancer, respectively (NCT02268825, NCT02375672). In a study of atezolizumab in combination with VEGF inhibition with or without chemotherapy, 7% of refractory patients showed response, 14% had stable disease for more than 24 weeks, and a total of 64% patients had stable response. Final data of this trial (NCT01633970) is estimated to announced at the end of 2018. In a very recent analysis, pembrolizumab in combination with mFOLFOX6 had shown efficacy in a phase II trial [82]. Of total 30 patients enrolled, one complete response had occurred in MSH tumor harboring patient and 53% of patients had partial response. The rate of grade 3/4 toxicity was 36.7% in combo arm versus 13.2% in chemotherapy only arm. There was no treatment associated death. Clinical activity was seen in patients with untreated advanced CRC including those with proficient MMR.

Another role of cytotoxic drugs on immune system is in metronomic schedules. Metronomic chemotherapy refers to the administration of chemotherapeutic agents at relatively low, minimally toxic doses, without a prolonged drug-free lag period. It allows for continued, low toxicity and more tolerable drug dosage applications for patients and had shown efficacy [71, 83]. Metronomic therapy was thought to primarily alter endothelial cells and acts via inhibition of angiogenesis [84]. Additionally, there is preclinical and clinical data that support the immune modulatory role of metronomic treatment [71, 83, 85, 86]. Metronomic cyclophosphamide was shown to be effective immunologically and decreasing circulating suppressor T-cell population in low doses whereas high dose applications resulted in depletion of whole lymphocyte population [83].

### 3.5. Combination of Immunotherapeutics with Targeted Agents

Cancer medications have developed in two parallel arms of science. Firstly, a deeper understanding of cancer biology, genetic drivers of carcinogenesis, and signal transduction pathways has led to the development of targeted agents for genetically chosen patients and has resulted in profound and rapid, albeit short-lasting, responses. Secondly, we have come to understand the different ways of tumoral escape from natural protective mechanisms of the body and have obtained immunotherapeutic drugs achieving more durable responses in various types of malignancies. Additional insights of targeted therapies and their effects on immunologic microenvironment of malignancies have served as a foundation for their combinational use.

The Mitogen activated protein (MAP) kinases (MAPKs) comprise part of the intracellular signaling cascade which is essential for signal transduction. Activity of MAPKs plays a crucial role in immune system activity in various steps. First of all, by taking part in cytokine production upon getting signal form toll like receptors, MAPKs are involved in the initial step of innate immunity. Secondly, MAPKs are important for differentiation of T lymphocytes in response to cytokines via binding to appropriate receptors. Additionally, T lymphocyte dependent cytotoxicity is correlated with MAPKs signaled apoptosis and enables the removal of damaged or transformed cells. Hence, the function and appropriate signaling by MAPKs are important for efficacy of immune system and serves as a promising therapeutic role.

Mitogen activated protein kinase pathway is also crucial for various melanoma cases for tumorigenesis. Inhibition of mutated BRAF and MEK has been investigated in many clinical trials and is now one of the most preferred treatments of BRAF mutated melanoma. Aside from clinical efficacy, BRAF and MEK inhibition leads to increased melanoma neoantigen expression, paradoxical activation of MAPK signaling on T lymphocytes, PD-L1 expression upregulation, and inhibition of suppressive cytokines [10, 38, 87, 88]. As the tumor progresses, neoantigen expression diminishes and immunogenic recognition also decreases. BRAF and MEK inhibition results in the reversal of recognition. Furthermore, in the early phases of BRAF/MEK inhibition, there is increased cytotoxic T-cell infiltration in tumor samples [87–89]. Clinical resistance to BRAF inhibitors has been found to be associated with increased PD-L1 expression on melanoma cells [90]. First of all, the combination of BRAF inhibitors with anti-CTLA-4 agents has been tested. A phase II study of sequential therapy with ipilimumab after vemurafenib showed that ORR was 30% with median OS 20 months [91]. However, it is also important to note that the Phase I trial of concurrent administration of ipilimumab and Dabrafenib (Dabra) was terminated early due to hepatotoxicity [92]. A second phase I/II study investigated the safety of Dabra + ipi doublet and Dabra + ipi + trametinib triplet therapy [93]. Severe colitis and intestinal perforation in triplet arm led to the early closure of this cohort. Anti-PD-1 and PD-L1 strategies with BRAF inhibition is also a popular combination for trials. Vemurafenib in combination with anti-PD-L1 agent atezolizumab (Atezo) was tested in the treatment of naive BRAF mutant melanoma cases, which yielded promising early results in RRs [94]. Triplet regimen with vemurafenib + cobimetinib + atezolizumab was tested, which yielded a 83% RR with cumulative 40% grade 3-4 adverse events. Based on these findings, a number of phase III trials have been designed and are currently underway (NCT02908672, NCT02902029). A randomized phase II study with Dabra + trametinib combination with pembrolizumab/placebo is now recruiting patients as a part of KEYNOTE-022 trial (NCT02130466).

Another target for combination strategies in melanoma is c-KIT. Preclinical data and murine models supported that c-kit inhibition results in augmentation of antitumor immunogenicity. A phase I dose escalation study confirmed a clinical response in a small subset of patients with imatinib and ipilimumab (NCT01738139) [95]. The clinical evidence obtained so far supports the clinical use of BRAF, MEK and c-KIT inhibitors with immune checkpoints with manageable toxicity.

Tumor vasculature not only is important for tumor growth and metastasis but also has a crucial role in tumor-immune cell interaction. Vascular endothelial growth factor (VEGF) modulates T-cell response and inhibits APC maturation and the migration of immune cells via endothelia [96–98]. Hodi et al. demonstrated the histologically proven augmentation of immune war with cytotoxic T cells and dendritic macrophages against tumor cells after ipi + Bevacizumab treatment [99]. Consequently, VEGF inhibition with checkpoint inhibitors may be an effective option for advanced tumors in order to increase the monotherapy response rates. Several clinical trials are currently investigating the clinical efficacy of this combination in mRCC, melanoma, glioblastoma, and NSCLC (NCT02210117, NCT02017717, NCT02210117, and NCT00790010).

Sunitinib is the VEGF receptor tyrosine kinase inhibitor that regulates signaling in tumor cells and vasculature. In an early phase I trial, Sunitinib/Pazopanib with Nivolumab was tested and demonstrated better antitumor efficacy compared to the use of single agent mRCC (NCT01472081). Furthermore, targeting agents of WNT pathway, AKT-mTOR signaling, and epidermal growth factor and its receptor inhibition may also be promising strategies for use in combination with IT.

Src family kinases (SFKs) promote cancer progression and are commonly expressed in nonsmall cell lung cancer (NSCLC). Johnson et al. investigated the efficacy of dasatinib in patients with advances NSCLC and showed that it had modest clinical activity [100]. Besides, dasatinib was shown to have immune boosting activity [101–103]. Recently, dasatinib is tested with nivolumab in “An Investigational Immuno-therapy Study to Test Combination Treatments in Patients With Advanced Non-Small Cell Lung Cancer” (FRACTION-Lung) trial (NCT02750514).

### 3.6. Combination of Immunotherapeutics with Radiation

Radiation therapy aids immune system in two ways. First of all, it does so via direct toxicity and killing of tumor cells, where antigens are released. Secondly, radiation works as an immune-adjuvant and the inflammatory microenvironment leads to the induction of immune response. Moreover, in murine models, researchers demonstrated that when radiation tumor infiltrating lymphocyte count is upregulated, suppressive CD 8 positive T-cells were abrogated [104]. The abscopal effect defined by Mole et al. refers to the tumor regression distant from the primary radiation field, which is clearly explained by the systemic immune stimulation effect of radiotherapy (RT) [105].

In various tumor models, experiments have demonstrated clinical efficacy of combination with IT [106–108]. CTLA-4 blockade showed synergy with RT [104, 109]. One of the mechanisms of resistance against radiation and anti-CTLA-4 agents was found to be related to T-cell exhaustion due to increased PD-L1 upregulation [110]. Therefore, the blockade of PD-L1 was tested in murine experiments, which yielded evidence supporting the use of combination of PD-L1 and RT [111]. In an analysis of melanoma patients who received RT after progression on ipilimumab, 62% of the patients had a abscopal type of response with 43% ORR [112]. Researchers tested pembrolizumab for head and neck cancer in the concomitant chemoradiation method with cisplatin at a fixed dose of 200 mg IV in every 3 weeks and reported that pembrolizumab with cisplatin is safe and has no deleterious effect on radiation or chemotherapy [113].

## 4. Conclusion

Novel developments in immunotherapy have led to a new era in cancer treatment. Immunotherapeutics, specifically PD-1 and anti PD-L1 antagonists, have shown to elicit important, durable, and safe responses in many tumor types that were once considered among the most desperate malignancies. However, the response rates for immunotherapies still remain modest and the most durable responses are observed only in a small subset of patients.

One of the key limitations of achieving broader responses in clinical trials is the complexity of the host immune system and its interactions with tumor cells. Besides, a more in-depth understanding of tumoral antigen production and recognition, as well as of the escape mechanisms from host immunity and the antitumoral death responses, is essential to overcome the major problems of immune-related drug development. Increased efforts in translational research will further shed light on anticancer drug developments, especially in the immunotherapy area, which will lead to a better understanding of the dynamic interactions between the host immunity and tumor cells.

In order to overcome restricted response rates and increase the number of patients who benefit from the treatment, approaches from precision medicine have been investigated, and predictive biomarker studies have been conducted. Besides these approaches, combinational applications of IT have been hypothesized as solutions for broader range benefits for patients and improved response rates. Among the combination strategies, a backbone with either PD-1 or PD-L1 antagonist drugs along with certain cytotoxic chemotherapies, radiation, targeted drugs, and novel checkpoint stimulators will be the most promising approaches in the future.

## Figures and Tables

**Table 1:** Selected Immunotherapeutics, mechanism of action, and major clinical therapeutic combinations.

	Mechanism	Potential combination strategy				
		Co-in.	Co-st.	targt.	CT	RT
Ipilimumab	anti CTLA-4	+	+	+	+	+
Tremelimumab	anti CTLA-4	+	+	+	+	+
Nivolumab	anti PD-1	+		+	+	
Pembrolizumab	anti PD-1	+		+	+	+
Atezolizumab	anti PD-L1	+		+		
Avelumab	anti PD-L1	+		+		
Durvalumab	anti PD-L1	+		+		

Co-in.: coinhibitory, Cost.: costimulatory, targt.: targeted, CT: chemotherapy, and RT: radiotherapy.
